# Analysis of stakeholders’ responses to the food warning labels regulation in Mexico

**DOI:** 10.1186/s12961-022-00922-2

**Published:** 2022-10-14

**Authors:** Regina Durán, Edalith Asmitia, Juan Rivera, Simón Barquera, Lizbeth Tolentino-Mayo

**Affiliations:** 1 Center for Nutrition and Health Research, National Institute of Public Health, Avenida Universidad 655, Santa María Ahuacatitlán, 62100 Cuernavaca, Morelos Mexico; 2 Population Health Research Center, National Institute of Public Health, Avenida Universidad 655, Santa María Ahuacatitlán, 62100 Cuernavaca, Morelos Mexico

**Keywords:** Front-of-pack labels, Nutrition label, Mexico, Policy, Food warning labels

## Abstract

**Background:**

In Mexico, the inclusion of a front-of-pack label in the Official Mexican Standard 051 (NOM-051 for its Spanish acronym) has been widely discussed for years by different stakeholder groups. In 2019, the NOM-051 modification project was proposed, which included front-of-pack warning labels. To be modified, it underwent a public consultation period where stakeholders sent their comments to be reviewed and considered. The purpose of this study was to analyse the stakeholders’ perspectives during the public consultation of the NOM-051 modification project.

**Methods:**

To assess perspectives, the 795 comments available on the National Commission for Regulatory Improvement website were analysed. Identity, expectations and demands were identified from each comment using content analysis in NVivo. In addition, frequencies and percentages were obtained.

**Results:**

Fifty-six percent of the comments were opposed to the NOM-051 modification project. Industry and business interest nongovernmental organizations were against it; they considered that their revenues and profits would be affected. Public interest nongovernmental organizations, academia and health professionals were in favour, stating that the changes proposed are fundamental to informing consumers and improving food choices, and an essential factor in reducing the prevalence of overweight and obesity. Stakeholders in favour expected that eating habits would improve, and demanded that the front-of-pack warning label suggested in the modification proposal be maintained.

**Conclusions:**

The comments opposed to the NOM-051 modification project were consistent with the literature, indicating that food industry stakeholders use all possible arguments to interfere in public health policies. The main issues used by the food industry to discredit the NOM-051 modification project coincide with those used in other countries to stop public health policies and with those used by the tobacco industry to avoid market regulations. On the other hand, those in favour looked after the interests of the Mexican population.

## Background

High consumption of products with high content of free sugars, total fat, trans fat and sodium is associated with an increased risk of obesity and noncommunicable diseases (NCDs) [1–3]. Mexico is one of the countries in Latin America with higher amounts of junk food sold (58 g per capita per day); junk foods are high in added sugar and saturated fats, and many are high in sodium [4]. In addition, obesity and NCDs have become a public health problem that has dramatically increased over the past few years. In 2021, 74.1% of the adult population in Mexico was living with overweight or obesity, 30.2% with hypertension and 10.6% with diabetes [4].

Front-of-pack labelling (FOPL) is recognized as a cost-effective regulatory measure to prevent and control obesity, NCDs and unhealthy diet [5, 6]. Different FOPLs are used worldwide, including warning labels, Health Star Ratings and traffic lights [7]. To address the increased rates of obesity worldwide, warning labels have been adopted in various countries [8–10].

Warning labels are a type of FOPL with text on the front of prepackaged foods and nonalcoholic beverages, informing consumers when a product contains excessive or high amounts of critical nutrients [7]. This FOPL is based on scientific evidence generated by researchers from different countries who are free of conflicts of interest [11–14].

**Fig. 1:**
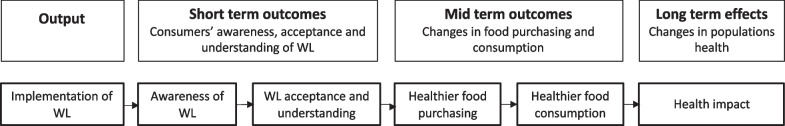
Logic model of the process by which warning labels impact purchase decisions and health outcomes

The way warning labels are expected to impact purchase decisions and health outcomes is shown in the logic model of Fig. 1. When the FOPL is accepted and understood, the population uses it to make healthier and informed purchase decisions [12–17]. Changes in the purchase may lead to changes in nutrient intake, which may have health impacts [5, 6, 18, 19].

In Mexico, the food industry voluntarily implemented the Guideline Daily Amounts (GDA) in 2010 [20]. Subsequently, in 2014, the Mexican government included it in the Official Mexican Standard 051 (NOM-051 for its Spanish acronym) for mandatory implementation [21]. However, this decision did not consider the scientific evidence demonstrating its disadvantages [20, 22–24] and the demands and recommendations of academia and civil society [25, 26]. In 2018, an opportunity arose to implement a scientific evidence-based FOPL—warning labels [27].

To implement the warning labels in Mexico, the NOM-051 needed to include them. For this, a modification project was prepared (see Table 1 for the main changes proposed in the project) and underwent a public consultation process held by the National Commission for Regulatory Improvement (CONAMER Spanish acronym) that lasted 60 calendar days. During this period, interested parties and stakeholders could make comments through the CONAMER webpage. Once the public consultation period for the NOM-051 ended, the Secretariat of Economy and the Federal Commission for Protection against Sanitary Risks (COFEPRIS Spanish acronym) held meetings with all interested sectors to study and address the comments received and made the necessary modifications to the project [28, 29]. In January 2020, the regulation was approved and published with minimal changes [29].

**Table 1:** Description of major changes proposed in the NOM-051 modification project

Section	Description of the NOM-051 modification project section
Front-of-pack labelling (warning labels and caption)	Included five labels for prepackaged food and beverages that exceeded the nutrient profile for energy, sugar, trans and saturated fats, and sodium that will be used under specific criteria: - *Excess calories* label will be used on (1) foods with 275 or more calories per 100 g, (2) beverages with 70 or more calories per 100 mL or (3) beverages with 8 calories or more of free sugars per 100 mL - *Excess sugar* label will be used on products whose sugar content is 10% or more of the total calories - *Excess saturated fats* label will be used if the product saturated fat content is 10% or more of the total calories - *Excess trans fats* will be used if the product’s trans fats content is 1% or more of the total calories - *Excess sodium* label will be used in (1) foods with 1 mg or more sodium per calorie, (2) beverages and packaged foods with 300 mg or more sodium and (3) noncaloric beverages with 45 mg or more sodium It also included the label “Contains sweeteners, avoid in children” for those prepackaged food and beverages containing sweeteners In total, it included six labels: Below all warning labels, the caption “Ministry of Health” was included
	Included the “Contains caffeine—avoid in children” warning caption to indicate when the prepackaged food or beverage contained caffeine
	Included six numeric warning labels, which represented the number of warning labels in the product. This was meant only for products whose main display area was smaller than 20 cm^2^ Below all numeric warning labels, the caption "Ministry of Health" was included
Nutrition declaration	A list of ingredients must appear on the label in descending order of proportions. Sugars must be declared, grouped by the list in parentheses with the specific names of all free sugars present in the prepackaged product
Endorsements	The label must not include a written, graphic or descriptive form indicating that the product, its use or ingredients or any other characteristic is recommended or endorsed by societies or professional associations
Advertising	If the product contains a warning label, it must not use characters, drawings, celebrities, gifts, offers, toys or contests, offers related to the price or content, visual/spatial games or social network advertisements on the product which encourage its consumption
Nutrition and health claims	Can use nutrition and health claims only when a product does not contain a warning label
Substitute product	In substitute products, included the caption “Substitute product”

NOM: Official Mexican Standard

Stakeholder participation in policy development and implementation is important [30, 31]. There is growing recognition of the need to incorporate their narratives as a component of the broad evidence base required to inform complex policy-making processes [31, 32]. The public consultation brings into the discussion the expertise and perspectives of the interested stakeholders. It ensures that regulations are designed and implemented in the public interest, includes various stakeholders and hears their views [33, 34]. However, this represents a risk due to the participation of people with a conflict of interest, who bias their opinion to protect their economic interests.

Different studies have analysed the response of specific stakeholder groups to public health policies [35–38] and public consultations on health policies [39–41]. Dorlach and Mertenskötter [41] analysed industry comments during the public consultation of Chile’s FOPL warning labels. The dominant argument used by the industry stakeholders was that the nutrition labelling regulation would violate international economic law [41]. Ares et al. [40] analysed the comments of industry and industry associations on the Uruguay public consultation for FOPLs, which included that the warning label violated international treaties and was not supported by scientific evidence, among others. To our knowledge, no analysis involving all the interested stakeholders has been undertaken on a FOPL public consultation. This paper aims to analyse stakeholders’ perspectives during the public consultation stage of the NOM-051 modification project.

## Methods

The Ministry of Economy reported that they received 5200 comments during the CONAMER public consultation period of the NOM-051 held from 7 October to 10 December 2019. Nevertheless, there only were 795 comments available on their website: http://187.191.71.192/portales/resumen/48142. We retrieved the comments from the website and analysed them. Repeated comments (*n* = 69, 8.7%), those that were not applicable (impossible to codify, incomplete or truncated responses) (*n* = 38, 4.8%) and those that did not refer to the NOM-051 modification project (*n* = 51, 6.5%) were eliminated. A total of 637 comments were considered and imported into NVivo 12 for their analysis.

### Analysis of comments

A mixed-methods analysis with a concurrent transformative design was carried out based on grounded theory and using content analysis as a technique. The stance of the key stakeholders was analysed according to what Clark defines as perspective, composed of *identity*, *expectations,* and *demands* [31]. *Identity* is composed of *judgement* and *justification*; for *judgement,* we identified whether the stakeholder was in favour of or opposed to the modification project; for *justification*, one or several reasons were identified to justify the judgement. *Expectations* are what the actor assumed or proposed would happen if the modification project were to go into effect. *Demands* are the actions the key stakeholders demanded.

The characterization of the stakeholders was based on their self-identification; those who did not self-identify were classified as individuals, not professionals. Descriptive statistical analysis was performed. We created a category tree from the analysis conducted by Monterrosa [39], and emerging categories were developed as the coding process progressed. Next, the team members coded 30 comments, which were randomly selected, and met with another member to verify their harmonization. The team members met periodically to discuss new codes, review them to ensure harmonization and merge similar codes. Two categories were created for *judgement*, 27 for *justification*, 21 for *expectations* and 28 for *demands*.

To analyse stakeholder perspectives, four cross-references were created in NVivo, where the codes of interest and types of stakeholders were used (if the aim was to identify *expectations*, the codes corresponding to *expectations* were selected). The coding references were taken into account, and percentages were obtained to obtain results. The cross-references were the type of actor along with (1) *judgement* (*n* = 637), (2) *justification* (*n* = 1088), (3) *expectations* (*n* = 467) and (4) *demands* (*n* = 865), and each of them was exported as an Excel matrix.

For some stakeholders, it was only possible to code two of the three perspective categories; others had more than one *justification*, *expectation* or *demand*, which is reflected in the difference in totals for each category. Codes representing < 2% of the coding references were gathered into a new code designated as “other not defined” for each category. To construct the narrative of the stakeholder groups, quotes were extracted from the principal codes.

## Results

### Characterization of the type of key stakeholders that participated in CONAMER's public consultation

Table 2 describes the characteristics of the stakeholders. Business interest nongovernmental organizations (BINGOs) had higher participation than those working for the public interest (PINGOs).

**Table 2:** Description of the stakeholders that participated in the public consultation of the NOM-051 modification project

Actor types	Actors	Description
Individuals (*n* = 348)	Nonprofessionals (*n* = 294)	Regular persons without any additional description
	Other professionals (*n* = 31)	Economists, lawyers and engineers
Industry (*n* = 113)	Food industry (*n* = 81)	Producers, buying and selling companies, marketers of juices, carbonated beverages, snacks, candies, chocolates, fruits, vegetables, natural and artificial sweeteners
	Balers (*n* = 12)	Producers of bags, packaging, plastic and cardboard containers
	Advertising agencies (*n* = 8)	Creating and advertising agencies
	Retailers (*n* = 6)	Self-identified retailers
	Others (*n* = 6)	Law firms and carriers
Academia (*n* = 98)	Students (*n* = 57)	University students
	Professors (*n* = 15)	Self-identified university professors
	Researchers (*n* = 14)	Self-identified researchers
	Institutions (*n* = 12)	Academic institutions
NGOs (*n* = 69)	BINGOs (*n* = 36)	Business interest nongovernmental organizations
	PINGOs (*n* = 33)	Public interest nongovernmental organizations
Health professionals (*n* = 23)		Doctors, nutritionists, psychologists and self-identified health professionals
Government (*n* = 9)	Organisms (*n* = 7)	Governmental entities and agencies
	Government officials (*n* = 2)	Federal deputies

NOM: Official Mexican Standard; NGO: nongovernmental organization; BINGO: business interest nongovernmental organizations; PINGO: public interest nongovernmental organization

### Perspectives of the key stakeholders who participated in CONAMER’s public consultation of the NOM-051 modification project

Of the total stakeholders that participated in the public consultation, 56% were opposed to the NOM-051 modification project; the BINGOs and the industry expressed it to a greater extent, arguing that it violated intellectual and industrial property and consumer protection. They considered that their revenues and profits would be affected and demanded a review of the NOM-051 modification project. Academia, PINGOs and health professionals were in favour of the NOM-051 modification project, stating that the warning labels provided clear and simple information to consumers and improved food choices. They expected that eating habits would improve and demanded that the FOPL suggested in the modification project be maintained (Tables 3 and 4).

**Table 3:** Judgement and justification by stakeholder group in CONAMER public consultation of the NOM-051 modification project

	Academia (*n* = 98) %	Health professionals (*n* = 23) %	NGOs		Industry (*n* = 113) %	Government (*n* = 9) %	Individuals (*n* = 325) %	Total (*n* = 637) %
			PINGOs (*n* = 33) %	BINGOs (*n* = 36) %				
Judgement								
In favour of	88.8	78.3	93.9	5.6	2.7	55.6	41.2	44
Against	11.2	21.7	6.1	94.4	97.3	44.4	58.7	56
Justification								
Income and job losses	0.8	–	1.6	2.3	5.2	–	4.2	3.3
Discrimination	–	2.4	–	9.0	3.6	–	5.4	3.9
Positive experience in other countries	6.2	7.3	13.2	0.8	–	–	2.7	3.5
Technologically unaffordable	–	–	–	5.3	1.6	–	3.0	2.0
Legal principles								
In accordance with human rights								
To information, food, childhood and health	10.8	19.5	27.1	–	–	15.4	3.0	6.4
Violation of human rights								
To information, food and health	0.8	2.4	0.8	2.3	11.8	7.7	3.0	4.9
To legal certainty and freedom of expression	–	–	–	7.5	19.6	7.7	1.5	7.0
Violation of international treaties	1.5	2.4	0.8	14.3	2.3	–	4.2	4.1
Violation of intellectual and industrial property and consumer protection	0.8	2.4	–	21.1	31.4	–	4.2	12.9
Health and well-being								
High obesity and NCD prevalence	20.8	7.3	8.5	–	0.3	30.8	4.2	5.5
It’s not the solution for the high obesity and NCD prevalence	–	2.4	–	4.5	1.3	–	6.3	3.0
Provides more information and improves food choices	23.9	22.0	17.1	0.8	0.3	7.7	12.0	9.7
Provides less information and complicates food choices	3.9	7.3	0.8	14.3	4.3	7.7	24.9	11.5
With scientific evidence support	20.0	12.2	21.7	2.3	1.0	15.4	7.2	8.4
Without scientific evidence support	6.2	2.4	0.8	12.0	15.7	–	9.0	9.6
Others not defined	4.6	9.8	7.8	3.8	1.6	7.7	5.1	4.4

CONAMER: National Commission for Regulatory Improvement; NOM: Official Mexican Standard; NGO: nongovernmental organization; PINGO: public interest nongovernmental organization; BINGO: business interest nongovernmental organization; NCDs: noncommunicable diseases

**Table 4:** Expectations and demands by stakeholder group in CONAMER public consultation of the NOM-051 modification project

	Academia (*n* = 98) %	Health professionals (*n* = 23) %	NGOs		Industry (*n* = 113) %	Government (*n* = 9) %	Individuals (*n* = 325) %	Total (*n* = 637) %
			PINGOs (*n* = 33) %	BINGOs (*n* = 36) %				
Expectations								
Human rights								
The right to information would decrease	–	–	–	8.7	17.8	–	6.7	11.7
The right to legal certainty would decrease	–	–	–	4.4	13.7	–	2.5	8.1
Health and well-being								
Obesity and NCDs would decrease	30.0	36.4	30.8	2.2	–	–	5.8	5.3
Product reformulation would decrease	10.0	–	–	30.4	2.5	–	15.0	8.7
Eating habits would worsen	3.3	18.2	–	13.0	17.4	25.0	19.2	16.2
Eating habits would improve	46.7	45.5	38.5	–	–	–	9.2	7.5
Economy								
Employment would decrease	–	–	–	2.2	19.1	25.0	10.0	13.0
Revenues and profits would decrease	–	–	–	15.2	27.0	25.0	15.8	19.8
Impact on commercial relations	–	–	–	8.7	0.4	–	5.8	2.6
Other not defined	10.0	–	30.8	17.4	2.1	25.0	11.7	7.0
Demands								
Technical changes to the NOM-051 modification project								
Maintain the sections of:								
Endorsements	6.2	8.2	11.1	–	–	4.2	1.9	4.1
Nutrient content claims	7.6	6.1	9.7	0.8	–	–	3.4	4.4
Warning caption	4.1	4.1	6.9	–	0.9	4.2	4.2	3.6
Portions	8.3	8.2	5.6	–	–	–	3.4	3.8
Advertising	15.2	8.2	14.6	–	–	4.2	9.8	8.6
Cutoff points for nutritional criteria	4.8	8.2	9.7	–	–	–	2.3	3.6
Warning labels	21.4	10.2	14.6	–	–	8.3	13.6	11.0
Nutrition facts label	10.3	8.2	9.7	0.8	0.9	4.2	3.4	5.2
Other not defined	1.4	6.1	2.1	7.4	2.6	4.2	2.6	3.2
Delete or modify the sections of:								
Endorsements, nutrition content claims and warning caption	2.1	–	4.2	13.1	9.5	8.3	2.6	5.2
Portions	0.7	2.0	0.7	4.9	0.9	–	6.4	3.1
Advertising	–	–	0.7	7.4	4.3	4.2	1.5	2.3
Cutoff points for nutritional criteria	1.4	2.0	0.7	5.7	7.8	4.2	5.7	4.2
Warning labels	2.8	2.0	–	13.1	5.2	12.5	9.4	6.4
Nutrition facts label	4.1	4.1	–	6.6	3.5	8.3	1.9	3.1
Modification of terms, definitions and numbering	2.8	–	3.5	8.2	3.5	12.5	4.2	4.3
Longer implementation times	0.7	2.0	–	7.4	3.5	4.2	5.7	3.6
Education and campaigns	2.1	6.1	2.8	9.0	5.2	8.3	6.0	5.2
Revision of the NOM-051 modification project	–	–	–	10.7	46.6	4.2	7.2	10.1
Other not defined	4.1	14.3	3.5	4.9	6.0	4.2	4.9	5.2

CONAMER: National Commission for Regulatory Improvement; NOM: Official Mexican Standard; NGO: nongovernmental organization; PINGO: public interest nongovernmental organization; BINGO: business interest nongovernmental organization; NCDs: noncommunicable diseases

### Perspectives from academia, health professionals and PINGOs

The main *justifications* given by academia, health professionals and PINGOs were that the NOM-051 modification project provided more information to consumers and improved food choices, that it aligned with the principles of human rights (information, food, childhood and health) and that it was supported by scientific evidence. Some examples of the arguments are presented below:

*...it is a fundamental measure to inform consumers, stop consuming these unhealthy food products and address these problems.* (Teacher, Judgement: in favour, Justification: provides more information to consumers and improves food choices)

*[It]... not only seeks to guarantee consumers’ right to health, but also the right to food, to information, and a proper application of the principle of best interests for children.* (PINGO, Judgement: in favour, Justification: in accordance with human rights)

*According to the World Health Organization, Front Warning Labels is a public policy strategy to address obesity. Because of this, I approve and welcome the fact that the health authorities put this proposal to modify the NOM-051.* (Health professional, Judgement: in favour, Justification: supported by scientific evidence)

Their main *expectations* were that eating habits would improve, and obesity and NCD rates would decrease.

*...it would change consumption patterns among Mexicans, discourage the consumption of ultra-processed products, and promote the consumption of less processed or natural foods, which are part of the traditional diet.* (PINGO, Judgement: in favour, Justification: eating habits would improve)

*Clearly, we would reduce the rates of diseases that affect our Mexico.* (Student, Judgement: in favour, Justification: obesity and NCDs would decrease)

They *demanded* that the modifications be maintained, including the warning labels, the prohibition of advertising on products with a warning label, and the use of nutritional facts labels on the back of the product.

*I strongly support using an octagonal “warning seal” as the format chosen for front-of-food labelling in Mexico.* (Teacher, Judgement: in favour, Demand: maintain warning labels)

*[Name of PINGO] supports the prohibition of the use of advertising elements on processed food packages (characters, celebrities, mascots, etc.).* (PINGO, Judgement: in favour, Demand: maintain the section of advertising)

*I consider that the proposal to group sugars, stipulated in Section 4.2.2.1.8, seems to be the most appropriate to facilitate reading.* (Health professional, Judgement: in favour, Demand: maintain nutritional facts label)

### BINGO and industry perspectives

The main *justifications* of the BINGOs and industry were that the NOM-051 modification project violated the legal principle of intellectual and industrial property and consumer protection, that it was not supported by scientific evidence and that it violated the human rights of legal certainty and freedom of expression.

*It exceeds and contradicts the Copyright and Industrial Property Law.* (Industry-advertising, Judgement: against, Justification: violation to intellectual and industrial property and consumer protection)

*The system of the nutritional front of pack labelling, as it is proposed, lacks scientific rationale of national or international reference.* (Food industry, Judgement: against, Justification: not supported with scientific evidence)

*The profiles on which the nutritional front of pack labelling system is based violates legal principles, such as legal certainty and equality, in addition to being technically incorrect.* (BINGO, Judgement: against, Justification: violation of human rights)

Their main *expectations* were that their revenues and profits would decrease, product reformulation would decrease and eating habits would worsen.

*This would ultimately lead to a decrease in investment, both domestic and foreign, as well as a collapse in the exchange of goods and services.* (BINGO, Judgement: against, Expectation: revenues and profits would decrease)

*The warning label for natural and artificial sweeteners proposed in the project would create a significant disincentive to reformulate sugar products.* (BINGO, Judgement: against, Expectation: product reformulations would decrease)

*Encouraging consumers to move away from packaged foods and beverages toward foods and beverages they produce at home, and toward foods and beverages with intrinsic nutrients, versus added nutrients, may not necessarily result in decreased calorie intake and could potentially increase it. And it could steer consumers away from needed vitamins that may be more prevalent in “vitamin-fortified” foods.* (BINGO, Judgement: against, Expectation: eating habits would worsen)

Their main *demands* were the revision of the NOM-051 modification project, the elimination or modification of the cutoff points established for nutritional criteria, and the elimination of the sections about nutrition content claims, endorsements and warning captions for caffeine and artificial sweeteners. Some of the arguments were as follows:

*Before falling into a legal dispute because of this series of contradictions and excesses, we recommend reviewing the NOM 051 modification project and making the necessary adjustments.* (BINGO, Judgement: against, Demand: revision of the NOM-051 modification project)

*It is vital to consider the nutritional profiles endorsed by the Codex Alimentarius since it is the body in charge of labelling, not PAHO [Pan American Health Organization].* (Food industry, Judgement: against, Demand: elimination or modification of the cutoff points for nutritional criteria)

*Its elimination is respectfully requested. The restriction of recommendations or endorsements by professional societies or associations does not consider that such organizations may have strict criteria for granting favourable opinions.* (Food industry, Judgement: against, Demand: elimination or modification of the section of endorsements, nutrition content claims and warning caption)

### Government sector perspectives

The main *justifications* of the government sector for implementing the front-of-pack (FOP) warning labels were the high prevalence of obesity and NCDs, human rights (to information, food, health and childhood) and support from scientific evidence. The following are some examples of the arguments used:

*In Mexico, the epidemic of overweight and obesity is affecting the health and well-being of all age groups. This epidemic is on the rise and requires measures that clearly and adequately inform the population about consuming processed products and beverages with high amounts of calories, sugars, salt, and saturated fats, all directly linked to obesity and cardiovascular diseases.* (Government sector, Judgement: in favour, Justification: high prevalence of obesity and NCDs)

*According to the Convention on the Rights of the Child (1989), children have the right to enjoy the highest attainable standard of health, and this requires a well-balanced diet, being able to enjoy a protective environment for their mothers, fathers, and other caregivers to provide them with healthy nutrition to ensure their healthy development; therefore, we consider that front of pack warning labelling on food and beverages is urgent.* (Government sector organizations, Judgement: in favour, Justification: in accordance with human rights)

*Everything points towards, as the academic literature indicates (Monterrosa et al. 2013), this being the way forward to respond to the public health emergency we are experiencing nationally and globally. *(Government sector agencies, Judgement: in favour, Justification: with scientific evidence support)

On the other hand, the main *expectations* of the government sector were mainly against the NOM-051 modification project. They stated that jobs, income and profits would decrease, and the population’s eating habits would worsen.

*A measure such as the one proposed would represent a severe drop in the sales of meat, fruit and vegetable products, bakery products, dairy products, edible oils, and other products made with inputs produced in Nuevo Leon, which would result in lower demand, lower income and even the loss of decent jobs for families in our region. *(Government sector, Judgement: against, Expectation: less employment)

*It is estimated that if the modification is carried out under the proposed terms, the indirect consumption of cane sugar in the main industrial consumers could be reduced by up to 50%, representing a decrease in the demand for cane sugar of approximately 900 thousand tons. Suppose a national average price of 11 000 pesos per ton of sugar is considered. In that case, this will represent a loss for the sector in the order of 10 billion pesos, that is, 16% of the total value of national sugar production.* (Government sector, Judgement: against, Justification: lower income and profits)

*The implementation of the modification project may cause doubts and confusion in the target population regarding the quality of food that the State is offering.* (Government sector, Judgement: against, Expectation: eating habits would worsen)

Among their main *demands*, they stated that the warning labels should be eliminated and that some terms, definitions and numbering should be adjusted.

*It is suggested to eliminate the paragraph [referring to numeral 4.5.3.4.3]. Warning labels [for small packages] that indicate a single number of seals a product would bear are not informative; they give an alert, but the consumer has no decision elements beyond a numerical value. It does not indicate which ingredients exceed the recommended dose or quantity.* (Government agencies, Judgement: against, Demand: elimination or modification of warning labels)

*Numeral 3.5: It is suggested to define the concepts differently, on the one hand, to define cane sugar and in a separate definition, high fructose corn syrup.* (Government agencies, Judgement: against, Demand: adjustments in terms, definitions and numbering)

### Individuals’ perspectives

Within their main *justifications*, some individuals mentioned that the NOM-051 modification project would complicate food choices and that its use is not scientifically supported.

*“These modifications to NOM-51 make the information unclear and very general. It intends to put seals that are similar or the same to products with very different characteristics but competes with each other. This will confuse the consumer.* (Male, Judgement: against, Justification: complicates food choices and provides less information)

*We believe that implementing Mexico’s Black Label Regulation is unnecessary because there is no conclusive scientific evidence to establish that consuming noncaloric sweeteners can affect health.* (Anonymous, Judgement: against, Justification: without scientific evidence support)

Contrarily, within the main *justifications*, we also found comments in favour, where they mention that it improves food choices by providing more information.

*I fully support the proposal to change and improve the labelling to really know the nutritional contribution of the products.* (Professional, Judgement: in favour, Justification: provides more information and improves food choices)

Their main *expectations* were against it, mentioning that it would worsen eating habits and decrease revenues and profits.

*Responsible consumption is discouraged rather than facilitated.* (Judgement: against, Expectation: eating habits would worsen)

*There would be great economic losses for a large part of the country's productive sector.* (Judgement: against, Justification: less revenue and profits)

The individuals in favour *demanded* that the proposed section regarding warning labels and advertising be maintained.

*Health authorities must protect vulnerable populations such as pregnant women, adolescents, and children. For the reasons above, a warning of the sweetener content in food is very pertinent.* (Nonprofessional individual, Judgement: in favour, Demand: maintain warning labels)

*I am in favour of prohibiting the use of such elements [characters, toys, etc.] in advertising directed, openly or not, to children or adolescents, as I consider it an abuse by companies.* (Nonprofessional individual, Judgement: in favour, Demand: maintain the modification in advertising)

On the contrary, individuals who were against it *demanded* the removal of the proposed warning labels.

*I would propose to continue with the front sugar content batteries [GDA] and not include octagons, which leads to a misunderstanding of what the product contains.* (Nonprofessional individual, Judgement: against, Demand: elimination or modification of warning labels)

## Discussion

The present research analysed stakeholder perspectives communicated during the public consultation stage of the NOM-051 modification project. This analysis shows the views of all the stakeholders that decided to participate during the public consultation. We found that the perspective of industry and BINGOs was opposed to the NOM-051 modification project, using arguments related to the drops in sales, how much the regulation would affect their sector, and that the proposal was inadequate and confusing for the population. Meanwhile, the perspective of academia, health professionals and PINGOs was in favour, stating that the change in FOPLs was fundamental to informing consumers and that it would be an important factor in reducing the prevalence of overweight and obesity. The government sector and individuals did not declare themselves to be in favour of or against it. However, they highlighted issues that coincided with those made by industry and BINGOs and with those made by academia, PINGOs and health professionals.

Among the main arguments issued by the industry and BINGO stakeholders against the FOP warning labels was that the modification project violated international treaties; this is in agreement with studies from other countries that analysed industry comments made during a public consultation for a FOPL [40, 41]. In terms of violation of commitments and international trade law, the industry’s arguments are not necessarily true; according to the World Trade Organization, the Agreement on Technical Barriers to Trade and the Codex Alimentarius, countries must take the necessary measures to protect the health of their population [42–44]. Another legal principle the industry and BINGOs mentioned is that it violates freedom of expression. The industry used the same argument to block legislation in San Francisco in which sugar-sweetened beverage commercials were required to have a warning label covering 20% of the advertisement [45].

Industry and BINGOs also mentioned that their revenues and profits would decrease. Other countries have analysed the arguments used by the industry to block public health policies, and the arguments are very similar to those found in the present study. In London, the food industry raised concerns about the negative economic consequences of implementing a public policy regulating the marketing of products high in critical nutrients [46]. When adding menu labelling was in play in California and in King County, Washington, the food industry raised concerns about implementation costs [37, 38]. In the case of income and profits, there is no scientific evidence supporting this; on the contrary, an evaluation of the effects of Chile’s FOPL on employment, salaries and gross profits of companies showed that there was no effect on these variables [47].

In the salient industry and BINGO issues, we can identify commonly used arguments as proposed by Mialon et al. [48]: (1) stress the number of jobs supported and the money generated for the economy; (2) highlight the potential burden associated with regulation (losses of jobs, administrative burden); (3) shift the blame away from the food industry—for example, focus on individual responsibility, the role of parents, physical inactivity; (4) fund research through academia, ghostwriters, own research institutions and front groups; and (5) litigate or threaten to litigate against governments, organizations or individuals [48]. Likewise, the World Cancer Research Fund International identified common tactics used by industry to interfere with the FOPL. It categorized them into four areas, namely (1) delay, (2) divide, (3) divert and (4) deny [49], which were identified as central themes in comments by the industry and BINGOs. Similarly, the tobacco industry developed a “playbook” to guide the behaviour of those who advocated against regulations in that market. The themes and tactics used to discredit the NOM-051 modification project and public health policies in other countries coincide with those used by the tobacco industry, suggesting that the private sector uses the same strategies to protect its interests [50, 51].

The stakeholders in favour of the regulation (PINGOs, academia and health professionals) noted that the regulation was supported by scientific evidence; this argument was also used in favour of the Uruguay FOPL regulation [52]. Stakeholders in favour also mentioned that it provides more information and improves food choices; a study on menu labelling in California, United States, found that the group promoting public health communicated an informed decision-making message [37], which is in line with the findings in this study.

Another salient topic mentioned was the importance of informing consumers about the excess of critical nutrients in ultra-processed products; in this sense, FOP warning labels guarantee the right to information. Based on scientific evidence, they also mentioned that it would improve eating habits and decrease obesity and NCD rates, which is in accordance with the main topics mentioned in the public consultation of the national policy for regulating the school food environment in Mexico [39].

On the other hand, the primary themes found in the government sector comments are contradictory, which may be due to several factors. First, the number of members in this stakeholder group is minimal (*n* = 9). Second, those who were opposed to the NOM-051 modification project used very similar arguments to those of the industry; this makes us think that there may be conflicts of interest within the government sector. On the other hand, those who were in favour also publicly supported the process, and apparently did not present a conflict of interest. Third, those opposed mainly expressed *expectations* and *demands*; those in favour only expressed *justifications* and *demands* without stating their expectations. This third aspect explains why *justification* is shown in favour and *expectations* against.

The main issues expressed by individuals coincided with those of the industry, suggesting the use of another mechanism described by Mialon et al. [48]: procuring the support of community and business groups to oppose public health measures.

Despite 56% of the comments against it and efforts by the industry and BINGOs to discredit the FOP warning labels in the public consultation process, the NOM-051 modification project was approved and published with minimal modifications (the warning label “Contains sweeteners, avoid in children” was changed to a warning caption containing the same information; endorsements were permitted when there was scientific evidence supporting it and as long as the product did not have a warning label; and the requirement for the caption “Substitute product” was eliminated).

Other studies have detailed the importance of advocacy coalitions in policy processes and highlighted that a strong coalition is key to advancing the policy-making process [37, 38]. In this case, we were able to recognize those stakeholders from different groups who strongly supported implementation and those who did not. We identified a political leader, academic institutions with significant trajectories, recognized researchers and PINGO leading institutions.

Even after the approval of the NOM-051 modification project, the industry tried to stop its implementation and also used the COVID-19 pandemic as an excuse to delay or suspend its implementation [53]. Despite this, Mexico went forward in implementing FOP warning labels. Further studies are needed to identify the mechanisms used by key stakeholders involved in developing public policies.

## Limitations and strengths

One of the limitations of this study is that 5200 comments were received during the NOM-051 public consultation. However, at the time of this study, only 15.3% were publicly available and were the ones that were analysed. All the comments were published in the Official Journal of the Federation (DOF) in a document responding to them. Nevertheless, for this paper, access to the complete commentary is necessary in order to determine judgements, justifications, expectations and demands. In addition, for some stakeholders, it was only possible to identify two of the four categories mentioned.

Among the strengths, all the stakeholder groups identified in the comments published on the CONAMER webpage were considered for the analysis. It should be mentioned that the comments were issued by people interested in the subject who benefited from or were harmed by its implementation, not by a sample selected by the researchers.

## Conclusions

Our analysis reveals that those who were opposed to the NOM-051 modification were looking out for their own interests, which is in line with what has been reported by similar studies. It also shows that the industry used multiple arguments to interfere in public health policies. The comments from academia and PINGOs were based on scientific evidence and demonstrated that they were informed on the topic; however, these key stakeholders’ participation in creating a public health policy has been poorly documented.

Therefore, a series of terms and conditions should be developed to guide participation in the public consultation process, including providing truthful data and declaring conflicts of interest. Transparency criteria should be established for information regarding the people who participate in public consultations, while maintaining the privacy of personal information. In addition, improved dissemination of public consultations is essential to increase participation and diversity of comments.

## Data Availability

The datasets analysed during the current study are available from the corresponding author on reasonable request.

## Abbreviations

BINGO: Business interest nongovernmental organization
CONAMER: National Commission for Regulatory Improvement
DOF: Official Journal of the Federation
FOPL: Front-of-pack label
GDA: Guideline Daily Amounts
NCDs: Noncommunicable diseases
NOM-051: Official Mexican Standard 051
PINGOs: Public interest nongovernmental organizations
